# Localized lymphedema after treatment for soft tissue sarcoma in the lower limbs: Comparison of improvement according to duration before lymphaticovenular anastomosis

**DOI:** 10.1002/ccr3.2278

**Published:** 2019-06-28

**Authors:** Shuhei Yoshida, Isao Koshima, Hirofumi Imai, Toshio Uchiki, Ayano Sasaki, Yumio Fujioka, Shogo Nagamatsu, Kazunori Yokota, Mitsunobu Harima, Shuji Yamashita

**Affiliations:** ^1^ The International Center for Lymphedema Hiroshima University Hospital Hiroshima Japan; ^2^ Plastic and reconstructive Surgery Hiroshima University Hiroshima Japan; ^3^ Plastic and Reconstructive Surgery Tokyo University Tokyo Japan

**Keywords:** localized lymphedema, lymphaticovenular anastomoses, medial knee, reconstruction surgery

## Abstract

Surgically invasive procedures involving the medial knee and inguinal regions can cause lymphedema. Lymphaticovenular anastomosis (LVA) could improve volume reduction and decrease the risk of cellulitis. However, it may be preferable to performed LVA as early as possible to achieve optimal results.

## 1 INTRODUCTION

It is still unclear whether surgically invasive procedures at the region of the medial knee possibly cause iatrogenic lymphedema. In this case report, we present two cases in which iatrogenic lower limb lymphedema developed after resection of soft tissue sarcoma. Lymphaticovenular anastomosis (LVA) was effective in both cases, but the effectiveness varied depending on the duration before LVA was performed. We encountered two patients who underwent resection of pleomorphic sarcoma in the medial knee region with subsequent covering of exposed soft tissue immediately after resection using flaps or skin grafting. Both patients had complaints of recurrent episodes of cellulitis and edema in the entire distal region of the affected lower limb from the knee, which had persisted even after tumor resection. We performed LVA procedures under local anesthesia. Circumferential measurements of the affected lower limbs were obtained pre‐ and postoperatively after LVA at 6 months for both patients. The improvement rates were then calculated. The lower limb lymphedema improved postoperatively with cellulitis prevention and volume reduction. However, the improvement rate differed depending on the duration before LVA. It is assumed that surgically invasive procedures in the medial knee region as well as the inguinal region could cause lymphedema, and LVA could decrease the frequency of cellulitis. However, it may be preferable to perform LVA as early as possible to achieve volume reduction.

Secondary lymphedema is a consequence of lymphatic failure resulting from trauma or parasitic infection and may also be iatrogenic.^1^ Breast cancer‐related lymphedema is seen in the upper limbs^2^ and gynecologic cancer‐related lymphedema in the lower limbs.^3^ Other malignancies, such as melanoma, sarcoma, lymphoma, prostate cancer, urologic cancers, and head and neck malignancies, can also cause lymphedema.^4^ Lymph node dissection in the groin is a well‐known cause of lower limb lymphedema,^5^ but it is still unclear whether surgically invasive procedures in the medial knee region can cause iatrogenic lymphedema.

In this report, we describe two cases of iatrogenic lower limb lymphedema following resection of soft tissue sarcoma. Lymphaticovenular anastomosis (LVA) was effective in both cases; however, the effectiveness of the procedure depended on how long the lymphedema had been present before LVA was performed.

## 2 PATIENTS AND METHODS

**Figure 1:**
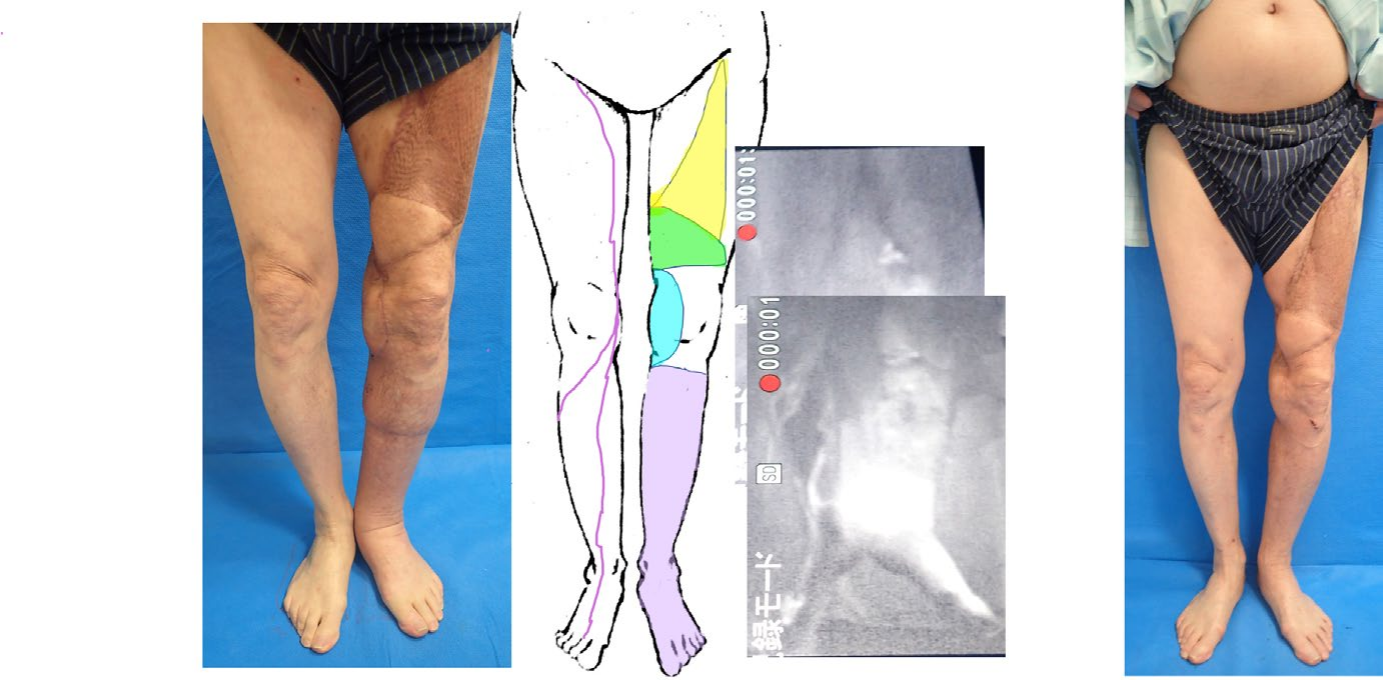
Case 1. Lymphedema and dermal backflow were observed in the entire distal region from the resected and reconstructed areas of the medial knee; linear patterns were observed in the right lower limb. Purple: indocyanine green lymphography. Green and blue: flaps. Yellow: skin graft

**Figure 2:**
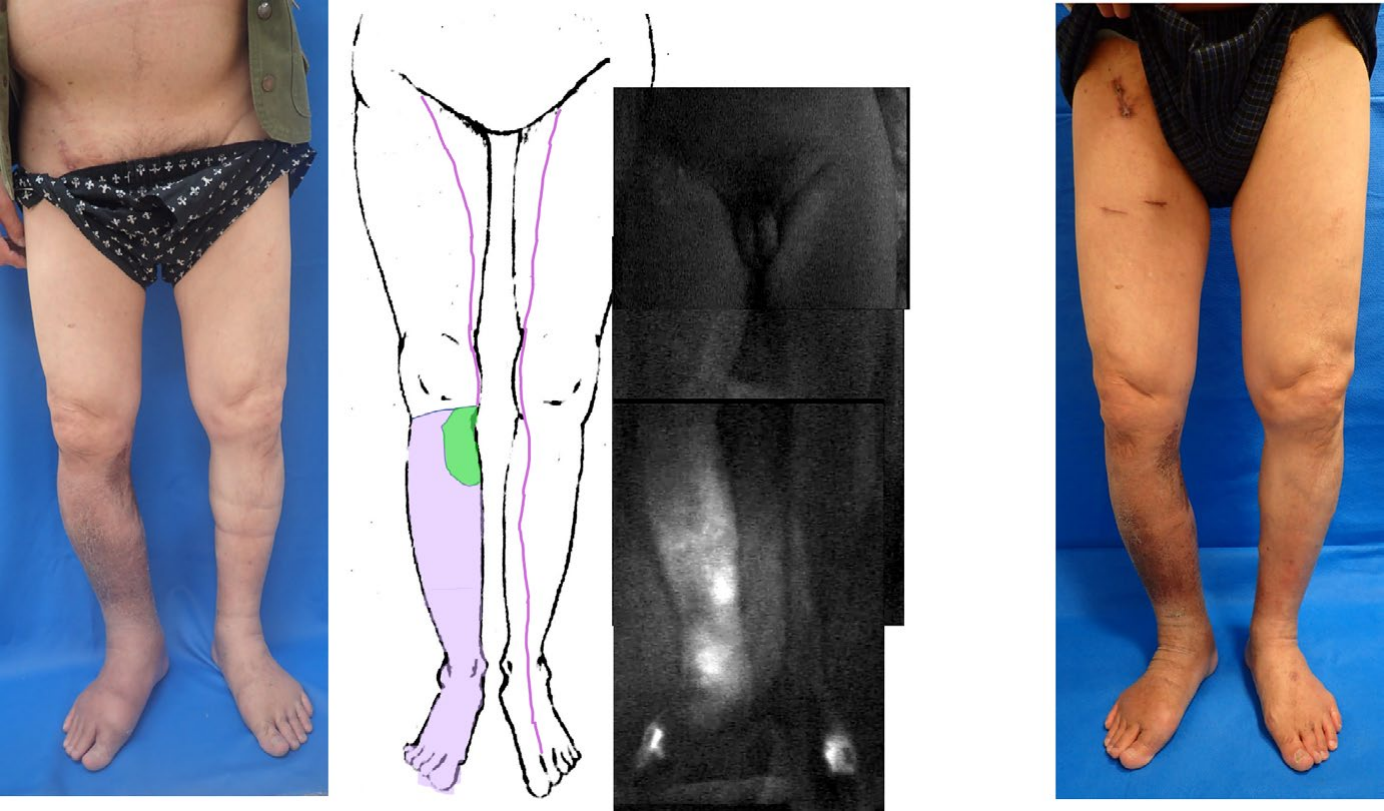
Case 2. Lymphedema and dermal backflow were observed in the entire distal region from the resected and reconstructed areas of the medial knee; linear patterns were observed in the left lower limb and right thigh. Purple: ICG lymphography. Green: skin graft

The two patients in this report underwent resection of pleomorphic sarcoma in the medial knee region with covering of the exposed soft tissue immediately after resection using flaps or skin grafting. Both patients had complained of recurring bouts of cellulitis and persistent edema in the entire distal region of the affected lower limb below the knee after surgery. They were referred to our department for treatment of their recurrences of cellulitis and persistent edema. Indocyanine green lymphography was performed for evaluation of lymph stasis. Dermal backflow was observed in the entire region distal to the resected and reconstructed area in the lower legs (Figures 1 and 2). Compression therapy had already been attempted using compression stockings in both cases. We performed the LVA procedure under local anesthesia. Circumferential measurements of the affected lower limb were obtained at five anatomic locations (10 cm above the knee, the knee, 10 cm below the knee, the ankle, and the foot) before and 6 months after LVA in the supine position after confirming that there was no cellulitis. Improvement rates were calculated by normalizing the difference in the circumferential measurement at each anatomic location before and after LVA by the preoperative circumferential measurement as follows:

([Preoperative circumferential measurement] − [Postoperative circumferential measurement])/(Preoperative circumferential measurement) × 100.

### 2.1 Case 1

The patient was a 71‐year‐old man who had undergone resection of soft tissue sarcoma and primary reconstruction of the left lower limb in October 2017. The tumor had measured 8 × 10 cm and an extensive amount of tissue had been resected, including part of the sartorius and gracilis muscles and the fascia of the vastus medialis. Two propeller flaps were raised from the calf region (a medial sural artery perforator flap) and the anterior thigh region (an anteromedial thigh perforator flap) after detection of a perforator on a color Doppler ultrasound scan. Mesh skin grafting was also performed on the surface of the quadriceps muscle. The perioperative course was uneventful. However, the patient was referred to our Lymphedema Center in February 2018 for treatment of persistent postoperative edema and repeated bouts of cellulitis in the left lower limb after surgery despite compression therapy with Jobst® opaque stockings (BSN Medical). His circumferential measurements in the affected limb were 45.5 cm at 10 cm above the knee, 39 cm at the knee, 34.5 cm at 10 cm below the knee, 24 cm at the ankle, and 23.5 cm at the foot. Body weight was 73 kg and height was 167 cm, giving body mass index (BMI) of 26.18. Three end‐to‐end LVAs were performed in the left lower limb in March 2018.

### 2.2 Case 2

The patient was a 71‐year‐old man who had undergone tumor resection and primary reconstruction in April 2012. The tumor had measured 10 × 10 cm and was extensively resected, with inclusion of the skin and fascia. Skin grafting was performed to cover the exposed area. The postoperative course was uneventful. However, edema of the right lower limb and bouts of cellulitis had persisted even after use of Jobst compression stockings. Therefore, he was referred to our Lymphedema Center in October 2017. Circumferential measurements in the affected right lower limb were 50 cm at 10 cm above the knee, 38.5 cm at the knee, 38 cm at 10 cm below the knee, 26 cm at the ankle, and 27.5 cm at the foot. Body weight was 75 kg and height was 165 cm, giving BMI of 27.55. Three end‐to‐end LVAs were performed in the right lower limb in November 2017.

## 3 RESULTS

### 3.1 Case 1

The lower limb lymphedema improved postoperatively without any need for compression therapy (Figure 1). The circumferential measurements in the affected left lower limb at 6 months after LVA were 45.5 cm at 10 cm above the knee, 31 cm at the knee, 29.5 cm at 10 cm below the knee, 19.5 cm at the ankle, and 21 cm at the foot; the rates of improvement in edema were 0%, 20.8%, 14.5%, 18.8%, and 10.6%, respectively (Table 1). Body weight, height, and BMI were the same as those before LVA. There has been no recurrence of cellulitis since this intervention.

**Table 1:** Comparison of improvement in circumferential length between the two cases

	Preoperative circumference (cm)	Postoperative circumference (cm)	Improvement rate (%)
Above knee (10 cm)			
Case 1	45.5	45.5	0
Case 2	50	50	0
Knee			
Case 1	39	31	20.8
Case 2	38.5	35	9.1
Below knee (10 cm)			
Case 1	34.5	29.5	14.5
Case 2	38	36	5.3
Ankle			
Case 1	24	19.5	18.8
Case 2	26	24.5	5.8
Foot			
Case 1	23.5	21	10.6
Case 2	27.5	24.5	10.9

Note

Improvement in case 1 was better than in case 2. The improvement rate was calculated by normalizing the difference in circumferential length for each anatomic location before and after lymphaticovenular anastomosis with the preoperative circumferential length.

### 3.2 Case 2

The lower limb lymphedema improved postoperatively, and compression therapy was continued for 3 months (Figure 2). The circumferential measurements in the affected right lower limb at 6 months after LVA were 50 cm at 10 cm above the knee, 35 cm at the knee, 36 cm at 10 cm below the knee, 24.5 cm at the ankle, and 24.5 cm at the foot; the rates of improvement in edema were 0%, 9.1%, 5.3%, 5.8%, and 10.9%, respectively (Table 1). Body weight, height, and BMI were the same as those before LVA. There has been no recurrence of cellulitis since the procedure.

## 4 DISCUSSION

These cases highlight two points, (a) lymphedema caused by invasive surgical procedures in the medial knee region can be treated by LVA and (b) the response to LVA may vary depending on the duration of lymphedema before LVA is performed.

Surgically invasive procedures involving the medial knee and inguinal regions may cause lymphedema, and LVA can decrease the frequency of cellulitis regardless of the decrease in volume. The lymphatic vessels transport not only interstitial fluid but also antigen information with dendritic cells to the lymph nodes. From the standpoint of acquired immunity, LVA is thought to create a bypass to the lymph nodes via which dendritic cells can transmit antigen information to T cells in the blood circulation.^6^

**Figure 3:**
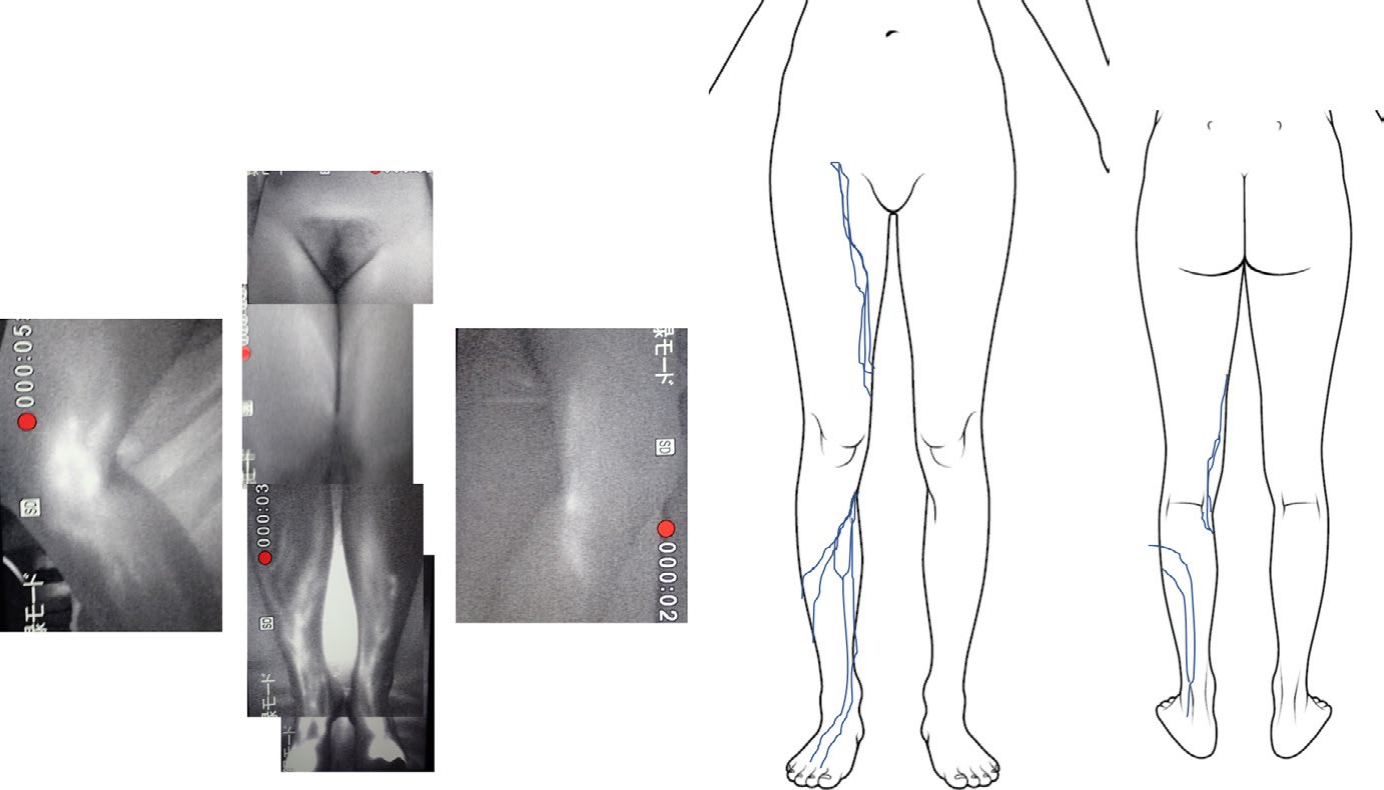
Lymphography (PDE system; Hamamatsu Photonics, Hamamatsu City, Japan) showing healthy lymphatic flow. The collecting lymphatic ducts can be seen aggregating at the medial knee

Secondary lymphedema can also be caused by invasive surgery. Lymph node dissection in the pelvic or inguinal region in particular is a well‐known cause of secondary lymphedema.^4^ It is reasonable to assume that surgically invasive procedures involving the knee region also induce secondary lymphedema as a result of damage to the lymphatics. However, except for a few cases, actual reports are very rare.^7^ From knowledge gained from our experience using indocyanine green lymphography, the collecting lymphatic ducts aggregate on the medial side of the knee (Figure 3). Given the wide range of motion at the knee joint, it is assumed that the complex of lymphatics around the knee area affects lymph flow in the entire lower limb.^8^ However, preoperative examination in the two cases presented here revealed that lymphatic vessels only accumulated below the knee. LVA cannot reduce volume when there is no accumulation of lymphatic vessels. We assume that this is why there was no change at the thigh level.

It is important to monitor patients for lymphedema after surgically invasive procedures to the medial knee region. Lymphedema not only causes edema but also increases the risks of infection and delayed wound healing in the affected limb, which is thought to be caused by localized immune deficiency in the area with lymphedema.^6^

### 4.1 Differences between the two cases

We regard the LVA procedure as successful in both cases because the volume of the affected limbs was reduced and there has been no cellulitis in either case since LVA. However, the improvement rate was better in case 1 than in case 2, likely because of the difference in the duration of lymphedema before LVA. In both cases, age, sex, and BMI, which are factors associated with exacerbation of lymphedema, were almost the same. The level and depth of injury was also almost the same in the two cases. Although the spread of invasion to the thigh was different, the lower edge of the level of invasion was on the same medial side of knee in both cases. The reticular system should have been more preserved in case 2; however, this patient showed less improvement. Therefore, we assume that the reticular system had almost no effect. Furthermore, the same surgeon performed the same number of LVAs in the same way. The only difference between the two cases was the duration of the lymphedema until LVA; therefore, we assume this factor could be an important determinant of the outcome after LVA.

Lymphaticovenular anastomosis is an effective surgical procedure,^9^ particularly in the early stage of lymphedema.^10^ LVA was performed 5 months after the onset of lymphedema in case 1, whereas more than 5 years had elapsed before LVA was performed in case 2. Accumulation of adipose and fibrous tissue tends to be progressive in patients in lymphedema, which contributes to the increasing size of the affected limb.^11^ Therefore, accumulation of adipose and fibrous tissue over a period of 5 years duration would have rendered volume reduction by LVA difficult in case 2. The difference in volume reduction between these two cases underscores the potential importance of performing LVA in the early stage of lymphedema.

## 5 CONCLUSION

Surgically invasive procedures involving the medial knee and inguinal regions can cause lymphedema, and LVA may decrease the frequency of cellulitis and improve volume reduction. However, it would be preferable to perform LVA as early as possible to achieve optimal results.

## CONFLICT OF INTEREST

None declared.

## AUTHOR CONTRIBUTION

YoS: performed as main surgeon of the work, writing and revising the article, and involved in management of all the work. KI: performed as main surgeon of the work and contributed to the concept, drafting the article, critical revision, and final approval of the version to be published. IH: performed as main surgeon of the work and participated in management of the patient. UT: participated in management of the patient. SA: participated in management of the patient. FY: participated in management of the patient. NS: participated in drafting the article, critical revision, and final approval of the version to be published. YK: participated in drafting the article, critical revision, and final approval of the version to be published. HM: participated in drafting the article, critical revision, and final approval of the version to be published. YaS: participated in drafting the article, critical revision, and final approval of the version to be published.
